# Differential Diagnosis of the Diseases of the Skin

**Published:** 1887-12

**Authors:** 


### DIFFERENTIAL DIAGNOSIS OF THE DISEASES OF THE SKIN.

*For students and practitioners.* By Condic W. Cutler, M. S., M. D. Pp. vi. and 139. New York: G. P. Putnam's Sons. Chicago: W. T. Keener. 1887.

The author's statement, that, "thanks to the untiring efforts of such students in dermatology as (here the names of four comparatively young Americans are given) and others, skin diseases are coming rapidly out of the obscurity in which they have been buried for years," etc., is very complimentary to the men whose names are given, but will cause any one to smile who happens to think who the "others" are who are so calmly ignored. But an unwise preface cannot spoil a good book, and this is a good book. The volume consists of Hebra's classification of skin diseses; some suggestions on taking the history of cases; and then follow, in tabular form, arranged in parallel columns, "The characteristic symptoms of those skin diseases which are liable to be confounded one with another." This tabular arrangement has been done both thoroughly and intelligently, and in its legitimate field the book will be found very useful. E. W.

